# Maternal positions during labor: Midwives’ knowledge and educational needs in northern Italy

**DOI:** 10.18332/ejm/136423

**Published:** 2021-05-20

**Authors:** Laura Garbelli, Viviana Lira

**Affiliations:** 1 Department of Clinical and Experimental Sciences, University of Brescia, Brescia, Italy; 2 Department of Obstetrics and Gynaecology, ASST Spedali Civili Hospital, Brescia, Italy

**Keywords:** mobility, labor, midwifery education, midwifery practice, maternal positions

## Abstract

**INTRODUCTION:**

Maternal positions and mobility during childbirth can have different and specific effects on labor and affect some birth outcomes. The aim of the survey is to investigate the knowledge and skills regarding maternal positions in labor among midwives and to consider the need of training.

**METHODS:**

A semi-structured questionnaire was distributed in August and September 2020 among midwives working in eight hospitals of Brescia, Northern Italy. The sample consisted of 115 midwives and data were analyzed using a quantitative, descriptive approach.

**RESULTS:**

The majority of the sample identified the general and specific benefits of maternal positions. Factors limiting the proposal of maternal positions were the context, the relationships with healthcare providers, the woman features, the fetal heart rate registration, continuous cardiotocography, amniotomy, episiotomy, operative vaginal birth, and epidural analgesia. Vaginal examination, the detection of uterine contractions, intrapartum ultrasounds, and ‘hands-on’ perineum technique were considered irrelevant by the participating midwives. The promoting factors were the presence of the partner, the telemetry, and the partogram with a section dedicated to positions. Nearly the totality of the sample considered appropriate to deepen the topic with training.

**CONCLUSIONS:**

Post-graduate courses are desirable to improve midwives’ skills. An educational toolkit is proposed to make the promotion of maternal positions more effective and appropriate. In order to improve midwifery intrapartum care, further research addressed to midwives of other settings appears essential to compare different training contexts, to expand the proposed toolkit, and to invest on midwifery practice and education.

## INTRODUCTION

The World Health Organization (WHO) recommends the importance of encouraging the adoption of mobility and an upright position during labor in women at low risk^1^. Moreover, the main international scientific societies state to avoid the supine position, most frequently associated with hypotension and fetal heart rate (FHR) abnormalities^2^ and to support women to assume whatever positions they find most comfortable^3-5^. It is important to highlight that there is not an ideal position that acts effectively and without distinction for every woman, but there are different positions that a woman is free to choose; during intrapartum care, no position should be imposed or forbidden.

Maternal positions and mobility play an important role in birth mechanics as they relate to some factors such as the pelvis type, the fetal position and attitude, uterine contractions, the gravity force, and woman’s preferences and emotional feelings^6,7^.

Several studies have been published evaluating the link between maternal positions and some childbirth outcomes, underlining their role in: promoting the physiological progression of labor^8^, facilitating the correct fetal positioning^9^, promoting fetal well-being^10^, making pain more bearable and increasing maternal satisfaction^11^, reducing perineal trauma^10,12^, influencing blood loss^10^, reducing the use of operative vaginal birth^8,13^, supporting partner involvement, and strengthening the empathic relationship between the couple and the midwife^11,14^.

On the other hand, another review^15^ reports that there are insufficient data to say anything conclusive about the effect of positions for the second stage of labor for women with epidural analgesia. However, women with an epidural should be encouraged to use whatever position they find comfortable in the second stage of labor^15^, as the WHO recommends^1^.

These conclusions, however, arise from moderate, low quality evidence with limitations and inconsistency of the included studies.

Indeed, the scientific evidence about the role of maternal positions and free movement in labor comes from individual surveys, systematic reviews, and meta-analysis. However, the results are generally fragmentary and not very solid due to the heterogeneity of the included studies and due to the difficulty of conducting randomized trials as women in labor do not maintain the same position for a long time. In addition, even the most rigorous reviews about maternal positions in labor often analyze the outcomes of the lithotomy position compared to all the others, generally defined as ‘vertical positions’, thus devaluing the specificity and indication of each single position. Indeed, gathering together all the positions in a unique group does not allow to highlight the peculiarity of each one and the adequacy of one with respect to another during a specific moment of labor. Albeit the difficulties emerged in conducting trials, it cannot be denied that maternal positions and mobility in labor are good obstetric practices, meant as veritable procedures that can have different and specific effects throughout labor.

The midwife, promoter of physiology, should know, be competent, and encourage the use of free positions during labor and birth^6,7^.

The aim of this survey was to investigate the knowledge and skills regarding maternal positions in labor among midwives and to describe the variables able to influence the proposal of maternal positions. Moreover, the need for specific training to deepen the topic was considered.

## METHODS

### Sample and data collection

The survey consisted of a quantitative, observational, and descriptive cross-sectional study, using a semi-structured online questionnaire as data collection method.

The prior authorization and ethical approval were obtained from the Nursing, Technical, and Rehabilitation Service (Servizio Infermieristico, Tecnico e della Riabilitazione Aziendale) of the birth centers included.

Individual informed consent was obtained from each participant, after the explanation of the study at the beginning of the questionnaire. Midwives were free to accept or decline participation. The online administration was realized by sending the questionnaire via email to the Association of Midwife’s Profession of Brescia for its distribution among registered midwives. In order to have a greater adherence and prompt collaboration, the tool was also sent to the Managers of the birth units.

Data collection was conducted by ensuring anonymity and respecting privacy, in accordance with GDPR 679/2016^16^.

The participating midwives practiced in the birth rooms of eight birth centers in Brescia, in Northern Italy, under the auspices of the National Health System. Brescia is a city in the region of Lombardy in Northern Italy. The city is the administrative capital of the Province of Brescia, one of the largest in Italy, with over 1.2 million inhabitants. A 2018 local report^17^ states that in this area there were 10154 births in the eight birth units. The Italian birth context has a classification system for levels of maternal care, comprising: I level Maternity Units providing care for low risk pregnancies or with minor complications, and II level Maternity Units dedicated to women with high risk pregnancies. Among the included birth centers in the study, six were a I level Obstetric Units while two were II level.

Inclusion criterion was Italian speaking midwives working in one of the hospitals in Brescia. Midwives who did not work in the birth room were excluded from the study as the first filter question aimed to limit the questionnaire to competent respondents.

The sample included 115 midwives based in the birth centers considered. Data were collected in August and September 2020.

### Instrument

The tool used for the survey was an anonymous, semistructured and ad hoc created questionnaire which was administered and distributed online using the Google Forms platform.

The questionnaire, consisting of 25 questions, was organized into three sections, realized after literature review and according to a logical, coherent sequence, functional to the survey’s objectives. The first section was dedicated to the personal assessment of knowledge and skills about maternal positions and of the context in which the midwifery care takes place. The second one analyzed the basic and technical-professional skills and knowledge related to the maternal positions. The third subdivision was based on the collection of personal and professional data of the included midwives.

More specifically, the analyzed variables included the personal assessment of midwives about maternal positions such as the level of personal knowledge, skills, and effective use of positions. Moreover, the knowledge of these general benefits was evaluated: promoting fetal progression and fetal positioning, containing maternal pain, reducing the risk of abnormal FHR, reducing labor’s length, preventing perineal trauma, making uterine contractions more regular and effective, encouraging partner involvement and the empathic relationship between the couple and the midwife. The references for general benefits were taken from the principal studies and reviews about the topic^6-14^.

Moreover, the specific benefits of some positions, namely squatting, hands-and-knees, kneeling, asymmetrical positions, walking, standing position and knee-chest, sidelying and semiprone on the same side as the fetal back were considered.

The references for the specific benefits were based on the research of Simkin et al.^6^, a milestone for midwives on the promotion of maternal position in labor.

In addition, the influence of the context (the care organization, the structure and facilities, the management of economical and working aspects) was considered. Even the relationship with other healthcare providers (such as midwives, gynecologists, anesthesiologists), the woman’s features and the presence of the partners were analyzed.

Among the clinical procedures conditioning the use of maternal positions during labor, the research evaluated the influence of cardiotocography, telemetry, intrapartum ultrasounds, the detection of uterine contractions, the auscultation of FHR, epidural analgesia, and the presence of partograms or protocols with a section dedicated to maternal positions. Even the vaginal examination, amniotomy, episiotomy, ‘hands-on’ method for perineal support and operative vaginal birth, were examined to verify their impact on the promotion of maternal positions.

Finally, the quality of received education and the need for specific training were considered.

As for the type of proposed questions in the instrument, the majority were closed multiple-choice, except for those that asked about the influence of some variables on the promotion of maternal positions. In this case, Likert scales were used. Respondents were asked to choose from a range of ordered responses: 1=‘Strongly uninfluenced’, 2=‘Uninfluenced’, 3= ‘Neutral’, 4= ‘Influenced’, and 5=‘Strongly influenced’.

### Pilot study

Before the distribution of the final questionnaire, a pilot study was conducted to verify the comprehension of the questions and to find any limits or errors of the proposed tool: it included the answers of 4 midwives. The pilot study allowed to modify the layout and to make their visualization through the used platform and their comprehension more immediate and clear; moreover a biased question was modified.

### Data analysis

Data were analyzed through the use of Microsoft Excel 2020; a descriptive analysis was conducted. To summarize the characteristics of the data set, descriptive statistics were used. As for multiple choice questions, measures of frequency were displayed. For Likert scale items, the codification used helped to divide variables that could influence the proposal of maternal positions into three groups according to sample’s frequencies: limiting, irrelevant, and promoting factors. Limiting factors were those that could be an obstacle to the promotion of maternal positions for the sample; irrelevant variables were those for which the majority of midwives expressed them as ‘neutral’, namely not influencing their proposal of positions; promoting factors were those that the sample considered facilitating the use of positions.

Bivariate descriptive statistics were used to verify differences in the influence of epidural analgesia and the presence of a protocol with specific section dedicated to maternal positions in each birth center. Also, the quality of the received education linked to the years of professional experience was assessed.

## RESULTS

### Sample features

The response rate to the questionnaire was 69.7%; 115 midwives replied among the 165 who worked in the birth rooms considered. The answer completion rate was 94.8%; the possibility that some specific questions remained unanswered was allowed as they were optional.

The sample included 113 females (98.3%) and 2 males (1.7%), with mean age 37.9 years and range 24–64 years. The majority of midwives (73.2%) had a Bachelor’s in Midwifery from a University (introduced from 2001), 11.6 % of midwives had a Midwife’s Diploma (introduced in Italy in 1997 and suppressed in 2000) and 15.2% said to have a Midwife’s qualification (before 1996). The education degrees are different according to the law in the year of achievement.

Of the sample, 81.1% obtained their qualification at the University of Brescia. Among the respondents, 22.6% attended 1st Level Postgraduate Programmes, 11.3% had a Master’s in Nursing and Midwifery Sciences (equivalent to the UK MSc) and 1.7% attended 2nd level postgraduate programmes. Postgraduate programmes provide additional specific expertise in order to increase professionalism: the 1st level can be attended after a Bachelor’s, and the 2nd level after a Master’s.

As regards non-academic postgraduate training, 52.7% attended clinical and professional courses dedicated to midwifery care during childbirth. Among these, 22.3% participated in specific training about maternal positions and mobility during labor.

The sociodemographic and education features of the sample (N=115) are shown in Table 1.

**Table 1:** Sociodemographic and education features of midwives in Brescia, Northern Italy in the study period August–September 2020 (N=115)

*Sociodemographic features*	*n (%)*
**Gender**	
Female	113 (98.3)
Male	2 (1.7)
**Age** (years)	
<25	5 (4.5)
26–35	39 (34.8)
36–45	46 (41.1)
46–55	17 (15.2)
56–65	5 (4.4)
**Education degree**	
Bachelor’s in Midwifery (from 2001)	82 (73.2)
Midwife’s Diploma (from 1997 to 2000)	13 (11.6)
Midwife’s qualification (before 1996)	17 (15.2)
**Place of degree achievement**	
University of Brescia	90 (81.1)
Other Italian universities	21 (18.9)
**Postgraduate academic education**	
1st Level Postgraduate programme	26 (22.6)
Master’s	13 (11.3)
2nd Level Postgraduate programme	2 (1.7)
None	77 (67.8)
**Postgraduate non-academic education**	
Courses on midwifery care during childbirth	59 (52.7)
Specific courses on maternal positions and mobility in labor	25 (22.3)
None	53 (47.3)

### Knowledge and skills of general benefits of maternal positions in labor

Concerning the individual assessment about the proposal of maternal positions in labor, 73% of the sample reported having a good or excellent knowledge of the topic; 73.1% presumed to have good or excellent skills of the topic; 76.5% affirmed that they use maternal positions in labor in a good or excellent way.

The results about the knowledge of general benefits of free positions and movements among the participating midwives are shown in Table 2.

**Table 2:** Knowledge of general benefits of maternal positions among midwives in Brescia, Northern Italy in the study period August–September 2020 (N=115)

*General benefits of maternal positions*	*n (%)*
Facilitating fetal progression through the birth canal	109 (94.8)
Promoting fetal positioning	105 (91.3)
Containing maternal pain	105 (91.3)
Reducing the risk of abnormal FHR and improve fetal oxygenation	85 (73.9)
Reducing labor’s length	67 (58.3)
Encouraging partner involvement	67 (58.3)
Reducing perineal trauma	62 (53.9)
Strengthening the empathic relationship between the couple and the midwife	50 (43.5)
Making uterine contractions more regular and effective	47 (40.9)

FHR: fetal heart rate.

More than 90% of the midwives identified the following as the three main objectives pursued with the proposal of positions and mobility: facilitating fetal progression through the birth canal, promoting fetal positioning, and containing maternal pain. Almost 75% of the sample believed that maternal positions can reduce the risk of abnormal FHR and improve fetal oxygenation. More than 50% of the midwives considered important to promote mobility in labor to reduce its length, to encourage partner involvement, and to reduce perineal trauma. Less than half of the sample believed that maternal positions can strengthen the empathic relationship between the couple and the midwife and can make uterine contractions more regular and effective.

### Knowledge and skills of specific benefits of maternal positions in labor

The knowledge of specific benefits was good for the indications of the following positions: the role of squatting position of taking advantage of the gravity force, enlarging the diameters of the pelvic outlet and relieving lower back pain (100%), of hands-and-knees as a position that would reduce the feeling of a premature urge to push before full dilatation (96.5%) and could prevent perineal trauma (64%) and of asymmetrical positions in facilitating internal rotation, and the correction of malposition or asynclitism (94.7%).

On the other hand, the knowledge was less accurate for the role of kneeling position in increasing the dimensions of the pelvic inlet and in resolving FHR alterations (19.7%). Even the knowledge of walking, standing, and knee-chest positions alternating to semiprone, as positions that could favor more effective contractions appeared to be imprecise (47.4%). The side-lying position, followed by the semiprone, on the same side as the fetal back, as a possible position for the correction of occiput-posterior fetal malposition was not well known by the responders (31.5%). These results are shown in Table 3.

**Table 3:** Knowledge of specific benefits of some maternal positions among midwives in Brescia, Northern Italy in the study period August–September 2020 (N=115)

*Maternal position*	*Effects*	*Correct response ( % )*
Squatting	Take advantage of the gravity force, enlarge the diameters of the pelvic outlet, relieve lower back pain	100
Hands-and-knees	Reduce the feeling of a premature urge to push before full dilatation	96.5
	Prevent perineal trauma	64
Asymmetrical positions	Facilitate internal rotation and the correction of malposition or asynclitism	94.7
Kneeling	Increase the dimensions of the pelvic inlet and to resolve FHR alterations	19.7
Standing, walking, knee-chest alternated to semiprone	Favor more effective contractions	47.4
Side-lying, followed by the semiprone, on the same side as the fetal back	Correct occiput-posterior fetal malposition in the advanced phase of labor	31.5

FHR: fetal heart rate.

### Factors influencing the proposal of maternal positions

As for the promotion of maternal positions in labor, 63.5% of the midwives reported that the context influences considerably the possibility of suggesting the positions to the woman in labor. However, 55.7% of midwives asserted that the relationship with other healthcare providers, such as midwives, gynecologists, anesthesiologists, can be an obstacle to positions’ proposal.

The features of the woman (such as culture, physical conditions, personality, behavior etc.) seemed to be a limiting factor in the use of positions for 67% of the sample and 57.9% thought that the presence of the partner, while recommending positions, plays a facilitating role. Moreover, 60.9% considered that continuous cardiotocography represents a brake to the use of positions. On the other hand, according to 72.1%, telemetry can positively affect the practice of maternal positions, while 47.8% reported that intrapartum sonography was irrelevant for the use of positions, and 21.8% considered it to be very helpful.

Regarding the influence of some clinical practices on the promotion of maternal positions, according to 47.8% the need to carry out a vaginal examination in free positions different from lithotomy does not represent a hindrance.

Moreover, in non-horizontal positions, the FHR auscultation appeared to be difficult for 74.6%, while the detection of uterine contractions was hard for 19.1%. According to 48.2%, the amniotomy affects the proposal of positions, while 29.8% considered it little or not at all limiting.

Of the midwives, 60% believed that the need to perform the episiotomy greatly impacts on the application of free positions; 52.7% considered that ‘hands-on’ technique for the prevention of perineal trauma is unrelated to the use of positions, whatever the adopted position is.

Of the sample, 33% considered epidural analgesia a limiting factor for the proposal of free positions. In two birth centers a clearer but contrasting opinion was recorded: for one birth place, epidural analgesia appeared to have considerable weight on the proposal of positions; in contrast, for another hospital it had an irrelevant role. Notably, 80.9% of the midwives reported that operative vaginal delivery deeply influences the use of free positions and mobility during labor. As for the influence of care tools on the proposal of mobility in labor, 61.8% of the midwives considered the partogram with a section dedicated to positions an essential instrument for their promotion.

In all, 73% reported that in their birth centers there was not a childbirth care protocol with specific references to the maternal positions that should be proposed in labor as an obstetric procedure.

As a consequence, according to the sample’s responses, the data analysis revealed that the factors that influence midwives’ skills can be divided into three categories: limiting, irrelevant, and promoting factors (Table 4).

**Table 4:** List of limiting, irrelevant and promoting factors affecting the proposal of maternal positions during labor among midwives in Brescia, Northern Italy in the study period August–September 2020 (N=115)

*Limiting factors*	*Irrelevant factors*	*Promoting factors*
Context	Vaginal examination	Presence of the partner
Relationship with other healthcare providers	Detection of uterine contractions	Telemetry
Features of the woman	Intrapartum ultrasounds	Partogram with section dedicated to positions
FHR registration	‘Hands-on’ perineum technique	
Continuous cardiotocography		
Amniotomy		
Episiotomy		
Operative vaginal birth		
Epidural analgesia		

FHR: fetal heart rate.

### Need of specific training among midwives

Regarding the education assessment, two-thirds of the sample reported that a Bachelor’s degree and post-graduate education did not provide a sufficient level of knowledge and skills on the use of maternal positions in labor. Almost the totality of the midwives (97.4%), although showing good knowledge of the importance of maternal positions, deemed appropriate to deepen the topic with a specific training.

## DISCUSSION

The majority of the sample identified the general benefits of maternal positions in labor. The knowledge of specific benefits was good for the indications of squatting, hands-and-knees and asymmetrical positions, while less accurate for other positions.

The data analysis also revealed that some factors may limit or facilitate the promotion of maternal positions in labor.

Among the most significant factors, the FHR registration appeared to be difficult for the majority of the sample. The concern of failing to get a good FHR recording quality might be due to the fact that signal loss, maternal heart rate acquisition, and artefacts are more frequent in some positions. Nevertheless, in low birth risk labors, this problem is overcome by the use of intermittent auscultation, which should be the recording method used routinely in physiological labours^3^.

The need to use continuous cardiotocography proved to be an impediment to the proposal of maternal positions. Actually, these data are in disagreement with the indications that suggest avoiding prolonged monitoring in maternal supine position, as it can determine aortocaval compression by the pregnant uterus, compromising placental perfusion and fetal oxygenation. Instead, side-lying, half-sitting, and upright positions should be preferred^18^.

Also, the necessity to perform an episiotomy was considered a limiting factor. However, one of the purposes of physiological intrapartum care should be reducing the inappropriate use of the episiotomy^3^ and the position adopted by the woman should not affect the execution of this procedure^14^. It is advisable to choose positions that facilitate the relaxation of perineal tissues and pelvic floor, avoiding situations in which the female genitals, too close to professionals’ hands, could lead to intervention.

The majority of the midwives believed that operative vaginal birth remarkably affects the proposal of maternal positions in labor, proving the difficulty or impossibility of performing an assisted birth in non-horizontal positions. However, there are recommendations for the prevention of operative vaginal birth through the use of specific maternal positions. Indeed, the recent RCOG 2020 guidelines^19^ recommend to encourage women to assume upright or lateral position in order to reduce the assisted birth rate.

As for the epidural analgesia, the sample results for this variable were not homogeneous. Anyway, women in epidural analgesia should be encouraged to move freely and to assume comfortable positions^1,4,15^ in order to support fetal head descent and mitigate hypotension, sometimes induced by used drugs.

Only a minority of the sample reported that intrapartum ultrasounds can be beneficial for suggesting positions. Even though the assessment and care plan to women are based firstly on the collection of clinical data through semeiotics, intrapartum ultrasounds can help to detect some useful information for the management of labor, such as the evaluation of fetal occiput position, of fetal station, and fetal progression^20^.

Among the promoting factors, telemetry was considered to favor the proposal of positions. The use of wireless sensors guarantees freedom of mobility to the mother during labor, rather than be restrained to bed in supine position, and should be therefore the preferable solution when available^18^.

Concerning good knowledge of the value of maternal positions in labor emerged in the study, however, a contradiction with the actual reality of childbirth was highlighted.

In Italy, there are no recent national data about positions during childbirth. Indeed, the latest national childbirth care report^21^, published in 2020, does not include information about the maternal position during childbirth. As regards the situation in Brescia, data regarding maternal positions at the moment of birth can be found in the childbirth document published annually by the healthcare district of Brescia. The most recent bulletin, published in 2019, states data referred to 2018^17^. The births in lithotomy position were 56.8%, with a considerable variability among the different hospitals: in 5 of 8 birth places in Brescia, about 8 in 10 women still give birth in lithotomy position. The supine position seems to be the preferred one, at least in the second stage of labor. Therefore, the WHO intrapartum care model^1^, which includes the adoption of maternal mobility and positions, is not properly applied.

### Educational proposals

The survey showed the need of training among midwives, that is desirable to be achieved through specific postgraduate courses of high specialization. This kind of training aims to answer to the needs of professional and cultural updating that, starting from the best scientific evidence available, allows the promotion of physiology through the use of non-instrumental semeiotics and maternal positions during childbirth.

**Figure 1:**
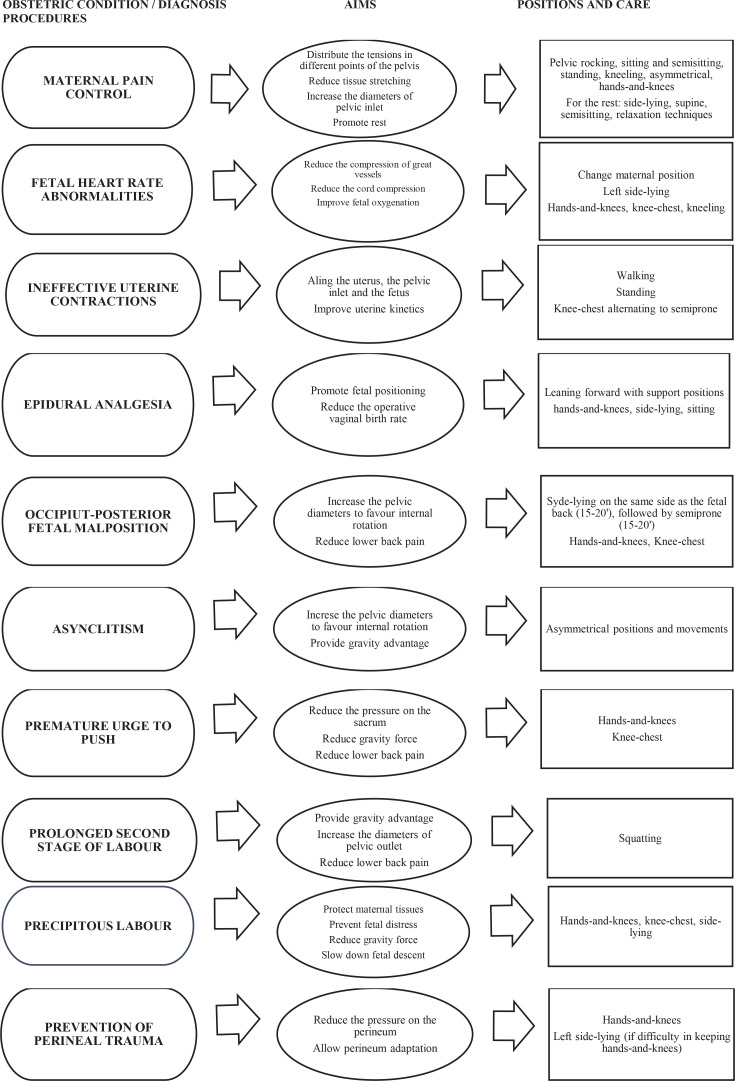
An educational toolkit about maternal positions during intrapartum care created after the results emerged among midwives in Brescia, Northern Italy in the study period August–September 2020 (N=115)

In order to improve midwives’ knowledge and competences, the model proposed by a group of midwives working in Bassano del Grappa Hospital (Veneto, Northern Italy)^22^ looks very functional. The document suggests some specific positions on the basis of the observed obstetric condition. Inspired by this flowchart, a similar tool is proposed (Figure 1). The model can be defined as a toolkit, namely an instrument whose objective is the implementation of evidence into clinical care^23,24^. The toolkit can make the promotion of maternal positions more effective, appropriate, and accessible. The toolkit, realized by taking into account literature data, recommendations of scientific societies, and considerations emerged in this study, was developed applying the midwifery management process^25^: starting from an obstetric condition or diagnosis, an objective achievable through the use of maternal mobility and positions is planned.

### Strengths and limitations

The topic of maternal positions in labor is largely debated. However, the majority of scientific articles focuses on birth outcomes and mothers’ experience related to mobility. Indeed, no previous studies were identified to assess midwives’ professionalism and confidence about the issue. In this way, this survey investigates knowledge and skills among midwives in order to implement and encourage midwives to update their abilities. Moreover, the educational toolkit proposed in this study (Figure 1) is an example of an original, well-designed instrument that could help midwives in the promotion of physiology and maternal positions during childbirth and it should be a starting point for future educational projects.

On the other hand, the main limitation of this work is related to the sample size: it involved only midwives working in birth rooms in the province of Brescia and therefore is not representative of the entire Northern Italian midwifery population. The sample is limited to the Brescia area and the results may be conditioned by the training received, which in 80% was achieved in the same university, with a lower possibility of comparison between different training contexts. Furthermore, the questionnaire used for data collection is not scientifically validated, although it was built with a defined methodology following literature review. An example of the imprecision of the questionnaire used could be the question about the presence of a partogram or protocols with a section dedicated to positions. In this case, these data were probably underestimated since in some cases the answers of midwives coming from the same birthplace were not in agreement. Probably the related question was not clearly formulated or not clearly understood.

## CONCLUSIONS

The study showed a good knowledge of the general and specific benefits determined by maternal positions and mobility in labor among the participating midwives. The evidence of limiting, irrelevant and promoting factors that influence the proposal of maternal positions emerged. Finally, most of the midwives expressed the need to enhance their knowledge and skills regarding the issue. In order to satisfy this, the proposed toolkit seems to be a valid instrument which should be interpreted as a starting point to invest on midwives’ education, to develop skills, to implement evidence-based practice into clinical care, and to encourage midwifery personnel to update their knowledge and abilities. In order to improve midwifery intrapartum care, further research addressed to midwives of other settings on a vast scale appears essential. This could allow to compare different training contexts, to expand the proposed toolkit, and to enhance and invest on midwifery practice.

## Data Availability

The data supporting this research is available from the authors on reasonable request.
